# Recognition of Emotion from Verbal and Nonverbal Expressions and Its Relation to Effective Communication: A Preliminary Evidence of a Positive Link

**DOI:** 10.3390/jintelligence11010006

**Published:** 2022-12-28

**Authors:** Jacob Israelashvili, Agneta Fischer

**Affiliations:** 1 Psychology Department, The Hebrew University of Jerusalem, Jerusalem 91905, Israel; 2 Department of Psychology, Social Psychology, University of Amsterdam, Nieuwe Achtergracht 129, 1018 WS Amsterdam, The Netherlands

**Keywords:** emotion recognition, emotional accuracy, empathy, language style matching, effective communication

## Abstract

Previous work has shown that emotion recognition is positively related to effective social interactions, but the mechanism underlying this relationship has remained largely unclear. Here, we examined the possibility that people who understand others’ emotions also talk to them using similar language. In the current study participants (N = 106) listened to emotional stories people shared from their own lives. They were later asked to recognize the storytellers’ feelings and finally provide written support messages. Perceivers’ ability to accurately recognize others’ feelings was assessed using the Emotional Accuracy Test (EAT), which uses naturalistic verbal and nonverbal emotional cues, and using two standard tests of nonverbal emotion recognition (GERT, RMET). The language of the expressor (target) was compared to the language of the supporter (participant) to quantify Language Style Matching, a proxy for effective communication. People who perform better in emotion recognition with verbal cues (EAT) also communicate their understanding and support using language similar to the expresser (*r* = .22, *p* = .02). This relation was insignificant for tests without verbal information (RMET, GERT). The result provides additional construct validation for the EAT and supports the view that understanding the emotions of others and communicating with them are two manifestations of a broader interpersonal skill.

## 1. Introduction

Individuals differ in their ability to recognize others’ emotions and this is related to how they manage social situations effectively. The importance of the ability to recognize emotions and its contribution to successful social functioning is articulated in models of Emotional Intelligence (e.g., Mayer et al. 2016; Scherer 2007) that consider emotion recognition and emotion management as two related abilities. This is also exemplified in findings that people who are good at understanding emotions report more satisfaction in their relationships and perform better in their jobs (see Côté 2022; Elfenbein et al. 2007 for reviews). Yet, how this proficiency in recognizing emotions is translated into successful social functioning has remained underspecified (Hall and Schwartz 2022).

Another factor that has shown to contribute to successful social functioning is similarity in conversations style. In a separate line of research, individual differences in mimicking the verbal style of conversation partners, such as repeating or referring to words and phrases that another person used (i.e., language style matching; Ireland et al. 2011) has been associated with effective social functioning, indicated for example by the quality of interpersonal relationships (e.g., Aafjes-van Doorn et al. 2020; Ireland et al. 2011), group cohesiveness and performance (Gonzales et al. 2010).

The question of the current paper is whether effective interpersonal functioning is related to emotion recognition and verbal mimicry. We assume that effective social functioning implies among other things that individuals are sensitive to others’ signals and can coordinate their own behavior with others. Thus, individuals who perform well in recognizing emotions might also use language that matches their conversation partner. However, extensive research on the relation between nonverbal mimicry and nonverbal emotion recognition did not find them to be reliably related. Individuals who perform well in nonverbal emotion recognition tests do not mimic others’ facial movements to a greater extent (Hess and Fischer 2014; see meta-analysis by Holland et al. 2021). Accordingly, one could assume that accuracy in recognizing emotions is unrelated to any form of (verbal or nonverbal) synchronization/mimicry. To our knowledge, there is no research on the relation between emotion recognition and the mimicry of the verbal communication style of one’s conversation partner. To clarify this ambiguity, we conducted the following study.

### The Current Research

The current study aims to examine whether people who identify others’ emotions accurately also communicate their understanding using language that matches the style of their conversation partner, and whether this also relates to more general emotion recognition ability. We administered the EAT (Emotional Accuracy Test), a recently developed and validated emotion recognition paradigm (labeled the [EAT]; for development study, see Israelashvili et al. 2020; for validation study, see Israelashvili et al. 2021). The EAT uses natural emotional expressions based on a series of videos in which real people share authentic stories about an emotional experience. In the current study, we asked participants to identify the storytellers’ feelings and subsequently to provide a written message of support to the target. In addition, we assessed accurate emotion recognition using two standard tests of *nonverbal* emotion recognition: the Geneva Emotion Recognition Test (GERT; Schlegel et al. 2014) and the Reading the Mind in the Eyes Test (RMET; Baron-Cohen et al. 2001).

The EAT differs from the GERT and the RMET in several ways, two of special importance: (a) In the GERT and the RMET, the emotional expressions are only nonverbal, while in the EAT, the emotional expressions are verbal and nonverbal; (b) In the GERT and the RMET, the basis of accuracy is the prototypical representations of emotional expressions, while in the EAT, the basis of accuracy is the agreement between the emotions experienced by the target vs. those perceived by another person (for a comprehensive comparison between the three recognition tests, see Supplemental Materials Table S2).

To assess verbal matching, participants were asked to provide a written message of support to one (randomly presented) target from the emotional accuracy test. The language of the participant (supporter) was compared to the language of the target (expressor) with which they told their story, to quantify Language Style Matching (LSM; Ireland et al. 2011). LSM is a metric that measures the degree to which two texts match in verbal styles. In practice, it calculates similarity in the rate that two people use various function words (e.g., articles, conjunctions). The focus on function words is related to the finding that they account for 60% of the words that people use in everyday communication (Gonzales et al. 2010; Ireland et al. 2011). The similarity between two individuals’ use of function words has been correlated with positive interpersonal outcomes (e.g., Ireland et al. 2011; Lord et al. 2015; Taylor and Thomas 2008; cf. Fusaroli et al. 2012), which suggests it may be used as an index of effective social behavior.

With the inclusion of different instruments to measure accurate emotion recognition, of which only one included verbal communication of an emotional story, we test whether the relation between identifying others’ emotions is related to emotion recognition more generally, or only to emotion recognition in a story telling paradigm. Inspired by models of emotion recognition as domain-general ability (Connolly et al. 2020; Schlegel et al. 2012, 2017; Lewis et al. 2016), we expected that the EAT would be most positively correlated because it pairs emotion recognition with verbal emotional communication.

## 2. Method

### 2.1. Participants

200 UK participants responded to a study advertised in Prolific Academic (“*View people in various situations and rate their emotions*”). Following our preregistered criteria (accessed on 29 September 2020: https://aspredicted.org/blind.php?x=kq67vw), we removed participants who provided low-quality data (e.g., failed attention check questions or their performance on one or more recognition test was below chance level); leaving a sample of 157 participants (M_age_ = 36, SD_age_ = 11; 64% female). Since 25 words are recommended as a minimum needed to allow the calculation of reliable linguistic metrics (Pennebaker et al. 2022), we excluded data of participants who provided fewer than 25 words in their messages of support. The remaining sample comprised 106 participants (M_age_ = 36, SD_age_ = 12; 65% female). A sensitivity analysis preformed in G-power suggested the analysis had a power of .80 to detect a medium effect (r = .265), with the standard criterion of α = .05. The Ethics Committee of the University of Amsterdam (EC 2020-SP-12183) approved the procedure of the study and we obtained informed consent from all participants.

### 2.2. Measures

Reading the Mind in the Eyes Test (RMET; Baron-Cohen et al. 2001). The test consists of 36 photos showing the region of the eyes of white individuals. Each image is coupled with four words that represents emotional state (e.g., alarmed, serious, bewildered, or ashamed; response options differ across the stimuli), and participants are asked to recognize the emotional state of the person in photo (0 = incorrect, 1 = incorrect). The total RMET score is determined by calculating the number of correct answers. In the current study the average RMET score was 26.5 (SD = 4.28).

Geneva Emotion Recognition Test (GERT; Schlegel et al. 2014). We used the short version of the GERT, which consists of 42 video clips, in which ten professional actors express seven negative and seven positive emotions according to their prototypical expression. Actors in each clip (duration 1–3 seconds) pronounce a series of syllables without semantic meaning, they are visible from their upper torso upward. Participants are instructed to select which the emotion (one out of the 14) that best describes the intended emotional expression of the actor (0 = incorrect, 1 = incorrect). The GERT score is determined by calculating the total of answers. In the current study, the average recognition score was 20.9 (SD = 4.42).

Emotional Accuracy Test (EAT; Israelashvili et al. 2020). In the EAT participants are presented with four video clips (2–3 min each; display order is random). In each video, a woman describes in English an emotional autobiographical experience (e.g., emotional distance from family, a parent being ill, etc.). Participants watch the videos and rate the intensity of ten emotions encapsulated in the experience of the target (anger, fear, disappointment, sadness, rage, worry, surprise, confusion, guilt and embarrassment; 0 = *not at all*; to 6 = *very much*). The targets’ own ratings were collected immediately after they shared the event. Specifically, targets watched their own videos and provided ratings of the emotions they felt in the video, using the same 10 emotions as presented to the participants. We used the absolute difference between participants’ ratings and the target’s own ratings to calculate accuracy across each of the 10 emotion rating scales (with larger absolute differences indicates lower accuracy) and averaged the score across all 4 targets. To simplify the interpretation of this index, we reversed the average absolute difference (−1* average absolute difference), such that a higher score represents better accuracy. The average absolute difference between the perceived vs. experienced intensity of emotions of the storytellers was 15.5 (SD = 2.66).

Language Style Matching (LSM; Ireland et al. 2011). We analyzed the language participants used when they provided support to a specific target and compared it to the language used by that target based on the transcripts of the emotional story they provided. Scores of LSM were obtained using the Linguistic Inquiry and Word Count (LIWC) software (Pennebaker et al. 2022). The metric of LSM has been developed by Ireland et al. (2011) and focuses on people’s similarity in the use of function words. There are nine categories of function words (i.e., articles, conjunctions, prepositions, quantifiers, adverbs, verbs, indefinite pronouns, personal pronouns, and negations). For each category, the metric calculates the similarity between two people in their respective use of terms related to a given category. Values are transformed to represent a range between 0 and 1 and averaged across all nine categories to yield a composite LSM score, with higher scores representing higher LSM. For more details, see the equation, explanation, and illustration we provide in the Supplemental materials (Table S1). Suggested levels for low and high LSM values are .60 and .85, respectively (Cannava and Bodie 2017). The average LSM score in the current sample was .68 (SD = .08).

### 2.3. Procedure

Participants completed the three emotion recognition tests (RMET, GERT, EAT) in a randomized order without time restrictions. After taking the emotional accuracy test, participants were randomly shown a photo of one storyteller and were led to believe that person would appreciate receiving a message of support (‘when recording the videos, this person indicated that they would appreciate knowing how people responded to their story. Please take a few moments to provide a written message of support’)^1^. Of note, participants completed the tasks as part of a larger test session, which addressed a different research question (i.e., whether performance on the Emotional Accuracy test relates to other existing measures of emotion recognition). This question was addressed in a separate manuscript (see Israelashvili et al. 2021). Here, we present only original analyses on correlations between each recognition test and language style matching. Thus, all the findings we report below are new and have not been previously published somewhere else.

## 3. Results

**Figure 1:**
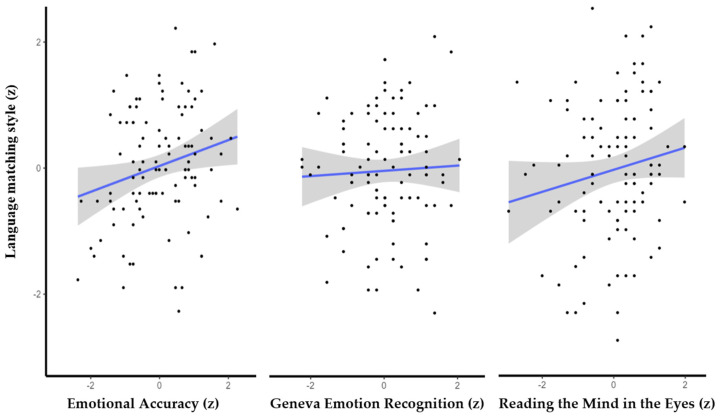
The simple correlation between language style matching and accurate emotion recognition on the EAT (**left**) and the GERT (**middle**), and the RMET (**right**). Note. Grey denotes 95% confidence intervals.

We used the Pearson coefficient to examine the bivariate directional (i.e., one-tailed) hypothesized correlations between performance on the three tests and the measure of language matching. Findings show that people who performed better on the emotional accuracy test (EAT) also articulate their support messages using language that matches their conversation partner. However, individuals’ performance on the RMET and the GERT was not significantly correlated with LSM scores (see Table 1 for statistical details and Figure 1 for illustration).

The positive relation between EAT and LSM remained significant when applying Bonferroni correction to account for multiple comparisons. Finally, for robustness check, we also examined the relation between EAT and LSM while statistically controlling for message length. The finding showed that individuals’ performance on the emotional accuracy test remained positively associated with the use of matching language, above and beyond variability in the message length (*r* = .2, *p* = .020).

## 4. Discussion

Both theory and research suggest a link between the ability to recognize emotions and effective interpersonal behaviors as two related constructs taping into border social and emotional abilities (Mayer et al. 2016; Scherer 2007; Schmid Mast and Hall 2018; Joseph and Newman 2010). However, the mechanism underlying this relationship has remained largely unclear (Hall and Schwartz 2022). Here, we examined one potential mechanism, namely that performance in understanding emotions might be associated with similarity in how people talk to one another. Findings suggest that people who accurately recognize others’ emotions might be those who match the language style of their conversation partner during communication.

However, the link between accurate emotion recognition and verbal matching style was only significant when accuracy was measured using the EAT, but not with the GERT or the RMET. These divergent patterns of associations across different recognition tests contradict our original expectations. We speculate that two factors might have contributed to these divergent associations. First, only the EAT includes *verbal* emotional expressions and thus has more direct commonality with an index of *verbal* mimicry. In fact, the written support was a direct response to the verbal emotional story of the target in the EAT. This implies that matching verbal communication is not related to the more general ability of emotion recognition. Second, the EAT operationalizes accuracy as an *agreement* between the emotions experienced by the target vs. perceivers, which coheres with the operationalization of language style as *matching* between target vs. perceiver uses of function words. On the contrary, the GERT and the RMET use the prototypical representations of emotional expressions as the basis of the accuracy score. Thus, it seems that the positive relation between verbal mimicry and emotion recognition can only be identified when recognition accuracy is based on matching criteria (i.e., EAT) rather than standardized criteria (GERT, RMET).

The current research provides the first evidence of a positive relationship between accurate emotion recognition and matching linguistic style. This relation between how people recognize and communicate verbal information is not trivial. A recent meta-analysis found that people high in *nonverbal* emotion recognition do not mimic others’ *nonverbal* facial movements to a greater extent (Holland et al. 2021 CE; see also Hess and Fischer 2014). Accordingly, one could expect that people with high vs. low ability to understand verbal and nonverbal emotional cues might not differ in verbally mimicking the style of their conversation partner. Nonetheless, the current study suggests that people who perform well in recognizing naturalistic emotional expressions also communicate with their conversation partners using similar language.

The positive relation between behavioral measures of emotion recognition (i.e., EAT) and verbal matching style (i.e., LSM) is meaningful for three reasons. First, it supports models of emotional intelligence (e.g., Mayer et al. 2016), which view understanding others’ emotions and effectively communicating with them as two components of a broader interpersonal skill. Second, people who recognize emotions well have more effective interpersonal interactions (e.g., Fischer and Manstead 2016; van Kleef and Côté 2022). The relation reported here between interpersonal accuracy (i.e., EAT) and matching communication style (i.e., LSM) suggests that emotion recognition might partially be translated into effective interpersonal functioning through verbal mimicry. Individuals who perform well in recognizing emotions can adapt their verbal communication to match their conversation partner, facilitating successful social interactions. Future research that will examine these variables together will be able to shed light on their potential mechanism. Third, previous research has found that LSM is associated with effective interpersonal behaviors (Ireland et al. 2011; Taylor and Thomas 2008). In the current study, we found LSM positively correlates with EAT. Taken together, it suggests that performance on the EAT indirectly associates with effective interpersonal behavior, further supporting the construct validity of the EAT.

Of course, the current research findings should be interpreted with caution. The positive relation we found was evident only in one of three recognition tests we examined, raising the possibility of incidental finding (i.e., type one error). To account for this concern, we adjusted the significance level to the number of tested correlations and found the positive relation (EAT, LSM) remained significant. Yet, a robustness check for the conclusion that emotion recognition tests using naturalistic (verbal and nonverbal) expressions correlate with the use of language matching style would be best achieved by replicating the present findings using a different set of stimuli or paradigms than used in the current study. Another limitation is that the messages of support were always requested after participants completed the EAT. We do not think that the fixed order of these two tasks has had a meaningful impact since the metrics used to calculate LSM and emotional accuracy use independent data sets. Still, future research should break this coupling to dispute its potential effect.

## 5. Conclusions

While the ability to recognize emotions and effectively interact with others is well-articulated in research and theory on emotional intelligence, the mechanism underlying this relationship has remained largely unclear. Here, we examined one potential mechanism: people who understand others’ emotions also talk to them in a matching language style–an index of effective communication. Our finding suggests that people who accurately recognize naturalistic emotional expressions of other people also communicate using language that matches the style of their conversation partner. We hope that more research characterized by ecological validity and behavioral indices, as employed here, will shed clarify whether how meaningfulness is a potential mechanism.

## Figures and Tables

**Table 1:** Description of the three recognition tests and Pearson correlation coefficients for their associations with language matching style (N = 106).

	Emotional Accuracy Test	Geneva Emotion Recognition Test	Reading the Mind in the Eyes
*Emotional Cues*	Verbal and nonverbal	Nonverbal	Nonverbal
*Basis of Accuracy*	Targets’ emotions	Prototypical expression	Prototypical expression
	***r*** (95% CI)	***r*** (95% CI)	***r*** (95% CI)
**Language Style Matching**	.215 * (.026, .390), *p* = .013	.072 (−.121, .259), *p* = .233	.111 (−.081, .296), *p* = .128

Note. * *p* < .05; CI = Confidence Intervals (lower, upper).

## Data Availability

The data presented in this study are available on request from the corresponding author. The data are not publicly available due to privacy.

## Note

1 We also collected additional measures for research questions unrelated to the present manuscript. Specifically, participants completed the IRI (Davis 1983) and the TIPI (Gosling et al. 2003), they were asked to rate their empathic responses toward the person in the video and finally to indicate whether they experienced similar event as their life. All these measures are fully described in our preregistration. Here, we focus on measures relevant to testing the hypothesis of the current manuscript.

## Supplementary Materials

The following supporting information can be downloaded at: https://www.mdpi.com/article/10.3390/jintelligence11010006/s1, Table S1. An illustration of the proportion of words related to a specific language style category used by the Expressor and two Supporters, based on the LIWC results within a given category; Table S2. Comprehensive comparison between the three recognition tests.
