# Supplementary material for: Recognition of Emotion from Verbal and Nonverbal Expressions and Its Relation to Effective Communication: A Preliminary Evidence of a Positive Link
Source: J Intell. 2022 Dec 28;11(1):6. doi: 10.3390/jintelligence11010006 (PMC9862275; doi:10.3390/jintelligence11010006)
Supplement: Supplementary file 1 [file jintelligence-11-00006-s001.zip › jintelligence-2078208-supplementary.pdf]

Supplemental materials

Details on the calculation of Language Style Matching (LSM)

The explanation here is partly adapted from Müller-Frommeyer, Frommeyer, & Kauffeld, 2019

This metric focusses on the similarity between two people in their use of function words. There are nine categories of function words: auxiliary verbs (e.g., to be, to have), articles (e.g., an, the), common adverbs (e.g., hardly, often), personal pronouns (e.g., I, they, we), indefinite pronouns (e.g., it, those), prepositions (e.g., for, after, with), negations (e.g., not, never), conjunctions (e.g., and, but), and quantifiers (e.g., many, few). The calculation of LSM scores required a series of analytic steps. First, in the numerator, the absolute value of the difference between the LIWC results within a specific language style category (C) for Speaker A (CA) and Speaker B (CB) is calculated, which is then—in the denominator—divided by the sum of CA and CB. In the denominator, .0001 is added to prevent the empty sets that would occur if the value for the category in question were 0% at each level of analysis. Then, the result of this fraction is subtracted from 1 (see equation below), resulting in a value between 0 and 1, with higher values indicating higher LSM in the respective categories. Finally, LSM scores for each of the function word categories are averaged to yield a composite LSM score, with higher scores representing higher LSM

$$LSM_{AB}(C) = 1 - \frac{|C_A - C_B|}{C_A + C_B + 0.0001}$$

An illustration of the process of language style analysis

To illustrate the process of language style analysis, below is the language of an expressor and two support messages, including their respective use of terms related to a given category and the resulting LSM scores.

> ***Expressor (target)****:* "I've found out that my father had heart problems, and he had to have open-heart surgery. They told my family that he only has about 50% chance of surviving the operation. When I first heard this, I think I was kind of shocked, and I didn't know what to say or what to do.."
>
> ***Supporter#1 (participant#1)****:* "My hope is that your father recovers completely from his cardiac issues and that he has sustained good health. In such times of difficulty, remember that you are more than capable of being a source of support for your father, the support he requires."
>
> ***Supporter#2 (participant#2)****:* "In difficult situations, one of the few things we can do is try and manage our own reactions. Your worry about your father is difficult, but he needs you. If you can be strong for him, you can turn to a friend with your worries - then you have support and don't pass any worry to your father. I hope all goes well for you."

Table S1. An illustration of the proportion of words related to a specific language style category used by the Expressor and two Supporters, based on the LIWC results within a given category.

	Personal pronouns	Indefinite pronouns	Articles	Prepositions	Negations	Common adverbs	Auxiliary verbs	Conjunctions	*LSM*
**Expressor**	18.52	9.26	1.85	12.96	1.85	5.56	14.81	7.41	
**Supporter#1**	15.91	6.82	4.55	15.91	0	2.27	9.09	4.55	**0.67**
**Supporter#2**	21.88	1.56	3.13	12.5	1.56	3.13	14.06	7.81	**0.81**

Table S2. Comparison between features of the three emotion recognition tasks.
The table here is adapted from Israelashvili, Pauw, Sauter & Fischer, 2021.

**Task**	**Stimuli**	**Emotional Cues**	**Emotional Expression**	**Basis of Accuracy**	**Choice Options**
RMET	Static pictures	Eyes (nonverbal)	Posed	Prototypical expression	Four (select one)
GERT	Dynamic videos	Voice, body and face (nonverbal)	Reenacted	Prototypical expression	Fourteen (select one)
EAT	Dynamic videos	Words, voice, facial and body movements (verbal and nonverbal)	Spontaneous	Targets' emotions	Ten (select all applicable, rate each using 0–6 scale)

Note. EAT, Emotional Accuracy Test; GERT, Geneva Emotion Recognition Test; RMET, Reading the Mind in the Eyes Test. An additional feature relevant to the stimuli is that the pictures of the RMET are all black and white, while the videos in the GERT and the EAT are all colorful. An additional feature relevant to the choice options is that in the RMET, every stimulus face is paired with four different choice options, while in the GERT and the EAT, all stimuli use the same fourteen (GERT) or ten (EAT) choice options.
